# Oncological safety of simultaneous transurethral resection of high-grade urothelial carcinoma of the bladder and benign prostatic hyperplasia

**DOI:** 10.1080/2090598X.2022.2142365

**Published:** 2022-11-11

**Authors:** Ben Valery Sionov, Matvey Tsivian, Pavel Bakaleyschik, Ami Abraham Sidi, Alexander Tsivian

**Affiliations:** a Department of Urologic Surgery, Edith Wolfson Medical Center, Holon, Israel; b Sackler Faculty of Medicine, Tel Aviv University, Tel Aviv, Israel; c Department of Urology, Wake Forest Baptist Health, Winston-Salem, NC, USA

**Keywords:** Bladder cancer, benign prostatic hyperplasia, lower urinary tract symptoms, outcomes

## Abstract

**Objectives:**

To examine the oncological safety of simultaneous resection of bladder tumor and prostate in the presence of non-muscle invasive high-grade urothelial carcinoma of the bladder (UCB).

**Materials and Methods:**

Between 2007 and 2019, 170 men with high–grade UCB who had a follow-up of at least 12 months were included in the study, including 123 with transurethral resection of bladder tumor (TURBT) only and 47 with simultaneous TURBT and transurethral resection of the prostate (TURP). We recorded and compared patients' clinicopathological parameters, recurrence, and progression rates during the follow–up period, as well as time to UCB recurrence in the bladder and the prostatic urethra/fossa.

**Results:**

Baseline demographic and pathological characteristics were comparable between the groups. At a median follow-up of 31 months in both groups, there were no significant differences in recurrence rates in the bladder and the prostatic urethra/fossa in either group (34.1% and 7.3% vs. 36.2 and 6.4%, p=0.402, p=0.363). No statistically significant differences were found between the two groups in terms of follow-up time, elapsed time to recurrence, or and progression in the bladder or prostatic urethra/fossa.

**Conclusions:**

Simultaneous TURBT and TURP in the presence of high-grade UCB appears to be oncologically safe in selected patients.

## Introduction

The co-occurrence of urothelial carcinoma of the bladder (UCB) and symptomatic benign prostatic hyperplasia (BPH) presents a unique treatment challenge. Worldwide, bladder cancer is the tenth most commonly diagnosed cancer overall and the seventh most commonly diagnosed cancer in men [1]. Lower urinary tract symptoms due to BPH are the most common benign condition associated with aging in men [2]. Therefore, it is not uncommon for a patient with UCB to also have symptomatic BPH [3]. Similarly, it is not uncommon to incidentally encounter a bladder tumor during cystourethroscopy in the setting of transurethral prostatectomy (TURP), which raises the clinical dilemma of whether to stage the procedures or perform simultaneous transurethral resection of the bladder tumor (TURBT) and TURP.

The approach to a patient with bladder cancer and symptomatic BPH is controversial. In many centers, there is a reluctance to perform concurrent surgery because of fears that tumor cells may implant in the exposed areas of the resected prostate tissue [4,5]. However, others have shown that simultaneous surgery for low-grade tumors is safe without increasing the rate of prostate recurrence [6,7]. In addition, reports demonstrate that earlier resolution of BPH symptoms decreases the recurrence rate and may positively impact a patient’s quality of life [8]. It is unclear whether the involvement of the resected prostatic urethra is increased by implantation in the presence of high-grade tumors, which are considered more aggressive and in which cells could theoretically have a higher propensity to seed.

This study aimed to evaluate the oncologic safety of simultaneous TURBT and TURP in high-grade UCB by evaluating recurrence rates in the bladder and prostatic fossa and comparing them with TURBT alone in a patient with concurrent symptomatic BPH.

## Materials and methods

Data were retrospectively obtained from a prospectively collected institutional registry of men who had undergone TURBT (group 1) or simultaneous TURBT and TURP (group 2) between March 2007 and December 2019. Only patients with pathologically proven high-grade NMIBC and BPH were included in this analysis.

Inclusion criteria for the study were simultaneous TURBT and TURP, histologically proven high-grade non-muscle invasive bladder cancer (NMIBC) and BPH, and preserved bladder during a year or more. Exclusion criteria were tumor involving the prostatic urethra, urethral stenosis, prior pelvic irradiation, incomplete TURBT due to excessive tumor volume, muscle-invasive bladder cancer (MIBC), and a follow-up period of fewer than 12 months. Only men with UCB were considered, and patients with other histopathology were excluded.

The reasons for concurrent surgery were the simultaneous presence of a bladder tumor and symptomatic BPH refractory to medical treatment in 35 patients, of whom six were with indwelling catheter, BPH requiring resection to access the bladder tumor in four, and the unexpected finding of a bladder tumor during cystoscopy in eight patients before TURP. Two patients suspected of bladder perforation during TURBT did not undergo TURP and were assigned to group 1 for analysis purposes. BOO was assessed using the International Prostate Symptom Score (IPSS).

All patients underwent standard TURBT using white-light cystoscopy and standard 26-Ch monopolar resection equipment with sterile water as continuous irrigation. When TURBT and TURP were performed simultaneously, TURP was performed after a complete TURBT and collection of all specimens. When TURP was performed, over-distention of the bladder was avoided to prevent damage to the bladder muscle. After completion of the surgery, a 22Fr urethral catheter was placed to allow continuous irrigation. The catheter was removed within 48 hours after surgery.

Histopathology reports were evaluated using the WHO criteria to determine grade and the TNM classification to determine stage [9]. In all cases, a re-TURBT was recommended 2–6 weeks after the initial surgery for all high-grade tumors (Ta/T1), except for the primary CIS. All pathological results were evaluated. All patients were offered intravesical therapy with either BCG or chemotherapy.

Our follow-up protocol included urinalysis, imaging (US/CTU) of the urinary system, cystourethroscopy, and urine cytology every three months for the first year, semiannually for the next two years, and then annually. If recurrence occurred, TURBT was performed for re-staging and risk stratification. Recurrence and progression were documented based on cystoscopy and pathologic examination results.

The primary endpoint was the rate of tumor recurrence and elapsed time to recurrence in the bladder and prostatic urethra/fossa in the presence of high-grade UCB. The secondary endpoint was the rate of postoperative urinary retention.

### Statistical analysis

Continuous variables were expressed as median values with interquartile range (IQR), and categorical variables were presented as numbers (percentages). Each group was analyzed and compared in terms of initial tumor characteristics, residual tumor rate at re-TURBT, overall and prostate urethra/fossa recurrence rate, time to the first recurrence, and progression rate during the follow-up period. Comparison between the two groups was performed using the Mann–Whitney U test, the chi-square test, or Fisher’s exact test when tables were too sparse. The incidence of recurrences was analyzed with the Kaplan–Meier method and the log-rank test. In addition, multivariate analysis using logistic regression was performed to identify risk factors for recurrence, including tumor stage, multifocality, concurrent carcinoma in situ (CIS), adjuvant intravesical instillation therapy, and simultaneous TURP. A P value of <0.05 was considered statistically significant. Data were analyzed using IBM SPSS statistics for Windows (version 23.0. Armonk, NY: IBM CORP).

## Results

**Figure 1.**
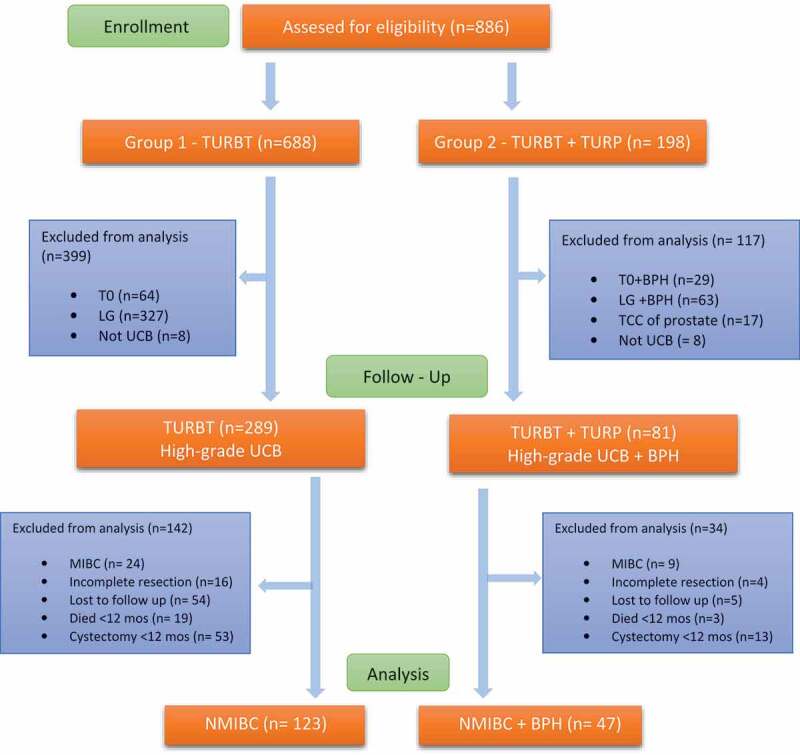
Flow diagram of study protocol.

Between March 2007 and December 2019, 1,121 patients underwent TURBT in our department, of whom 886 were men. of these, 367 men were diagnosed with high-grade UCB. The flow diagram of the study is shown in Figure 1. We included 123 patients who underwent TURBT only (group 1) and 47 patients who underwent simultaneous TURBT and TURP (Group 2). Patient’s age, tumor size, multifocality, sub-stratification histopathologic staging reports, follow-up, and percentage of adjuvant intravesical therapy were comparable between the two groups (Table 1).

TURBT – transurethral resection of bladder tumor; TURP – transurethral resection of bladder prostate; HG – high-grade; LG – low-grade; MIBC – muscle invasive bladder cancer.

**Table 1.** Paitents demographics and clinicopathological parameters.

	TURBT (N = 123)	TURBT+TURP (N = 47)	P value
Age (years), median [IQR]	73 [66–81]	78 [72–84]	0.162
Tumor Multifocality, n (%)			0.507
Solitary	66 (53.7)	26 (55.3)	
Multifocal	57 (46.3)	21 (44.7)	
Tumor size (cm), median [IQR]	2.5 [2–4]	2 [1–3]	0.105
<3, n (%)	84 (68.3)	36 (76.6)	
>3, n (%)	39 (31.7)	11 (23.4)	
T stage, n (%)			0.305
Tis (CIS)	4 (3.2)	3 (6.3)	
Ta HG	20 (16.3)	8 (17.0)	
T1 HG	99 (80.5)	36 (76.7)	
Concurrent CIS, n (%)	31 (25.2)	12 (25.7)	0.578
LVI, n (%)	22 (17.1)	7 (14.9)	0.322
Risk stratification, n (%)			0.347
Intermediate	6 (4.9)	3 (6.4)	
High	117 (95.1)	44 (93.6)	
Residual disease in re-TURBT, (%)	32.9	23.5	0.151
Induction Intravesical therapy, (%)	86.2	87.2	0.429

Group 1: TURBT only. Group 2: simultaneous TURBT + TURP

*HG* High-grade. *CIS* Carcinoma in situ. *LVI* Lymphovascular invasion

Concurrent CIS with high-grade UCB was 25.2% in group 1 and 25.7% in group 2. The median patient IPSS and weight of resected prostate tissue in group 2 were 22 (IQR 18–22) and 20 g (IQR 13–32), respectively. Re-TURBT was performed in 82 (66.6%) patients and 34 (72.3%) patients in group 1 and 2, respectively, and the residual tumor was found in 32.9% and 23.5% of patients in groups 1 and group 2, respectively (p = 0.151). 106 (86.2%) patients in group 1 and 41 (87.2%) in group 2 received intravesical therapy. The median time of administration of adjuvant intravesical therapy was within six weeks postoperatively in both groups (p = 0.088). Patients who underwent TURBT only were significantly more likely to experience postoperative urinary retention due to BPH (8.8% vs. 3.6%, p = 0.044). Of the 13 patients with urinary retention in group 1 under tamsulosin treatment, 3 (23%) patients failed to void on several trials (<4 weeks) and underwent TURP.

**Figure 2.**
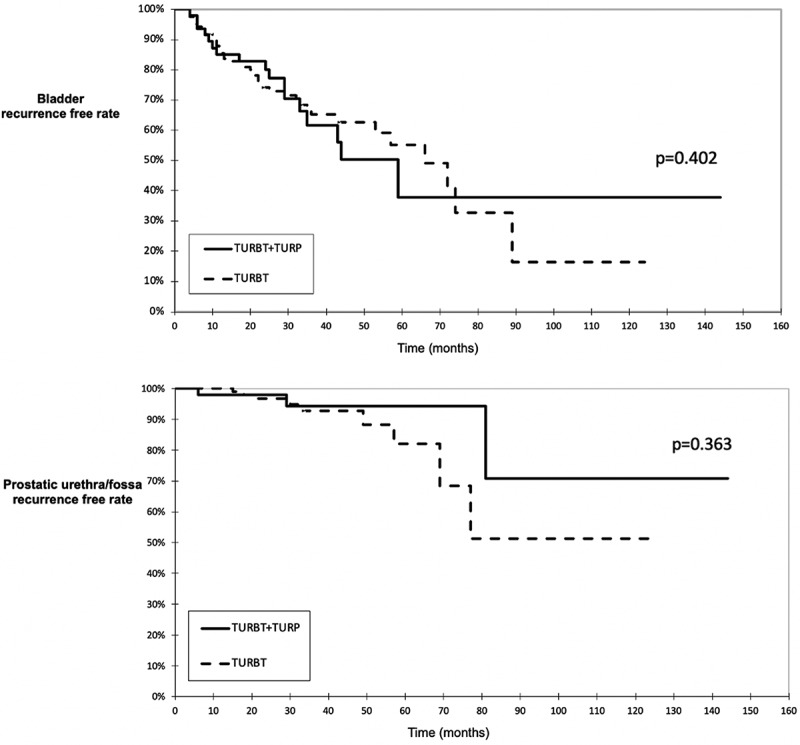
Multivariable analysis of bladder recurrences and prostatic urethra/fossa recurrences.

The median follow-up time in groups 1 and 2 was 33 and 31, respectively. During follow-up, overall and prostatic urethra/fossa recurrence rates were similar in both groups. They, developed in 43 (34.9%) and 9 (7.3%) patients in group 1 and in 17 (36.2%) and 3 (6.4%) patients in group 2, respectively. The recurrence rates are shown in Figure 2. The time to recurrence in the bladder and prostatic urethra was shorter in group 2 without being statistically significant: 16.5 months and 33 months in group 1 versus 17 and 29 in group 2 (p = 0.258). In twelve patients, six (66.6%) in group 1 and three (60%) in group 2, in whom prostate recurrence occurred, trigone, behind the bladder neck, or at the bladder neck were the most common locations (Table 2).

**Table 2.** Follow-up results.

	TURBT	TURBT+TURP	P value
No. pts	**123**	**47**	
Post-operative AUR, n (%)	13 (8.8)	2 (3.6)	0.044
Follow-up (mos), median [IQR]	33 [22–51]	31 [24–45]	0.131
No. recurrence in the bladder (%)	43 (34.9)	17 (36.2)	0.402
Time to 1^st^ rec in bladder (mos), median [IQR]	16.5 [9.5–32.2]	17 [8.5–34]	0.258
No. recurrences in PU/fossa (%)	9 (7.3)	3 (6.4)	0.363
Time to 1^st^ rec in PU/fossa (mos), median [IQR]	33 [19.5–63]	29 [6–81]	0.449
No. Progression (%)	15 (12.2)	7 (17.1)	0.327

Group 1: TURBT only. Group 2: simultaneous TURBT + TURP

*AUR* Acute urinary retention. *PU* Prostatic urethra

Multivariate analysis is shown in Table 3 and indicates that tumor multifocality was associated with recurrence in both groups (odds ratio 2.41 in group 1 vs. 2.72 in group 2). In contrast, a higher T stage was associated with a higher risk of prostate recurrence (odds ratio, 1.70 in group 1 vs. 3.54 in group 2). The rates of progression to MIBC were similar in both groups, 12.2% in group 1 vs. 17.1% in group 2 (p = 0.327). Of these, 5 and 3 patients from group 1 and group 2 underwent radical cystoprostatectomy, respectively.

**Table 3.** Multivariable analysis of recurrence.

Variable	Bladder recurrence	Prostatic urethra/fossa recurrence
T stage	1.7023 (0.7514–3.8569)	3.5448 (1.2778–9.8335)
TURP	1.0542 (0.5230–2.1251)	0.8636 (0.2234–3.3386)
Multifocality	2.4126 (1.3323–4.3691)	2.7210 (1.0689–6.9268)
Concurrent CIS	1.0370 (0.5448–1.9736)	0.4363 (0.1430–1.3311)
LVI	1.6074 (0.5082–5.0838)	0.7521 (0.1802–3.1386)
Adjuvant Intravesical therapy	0.1928 (0.0678–0.5482)	0.2748 (0.0923–0.8184)

Data are presented as odds ratio (95% confidence interval)

*CIS* Carcinoma in situ; *LVI* Lymphovascular invasion.

### Discussion

The co-occurrence of urothelial carcinoma of the bladder (UCB) and symptomatic BPH presents a unique treatment challenge. In many centers, there is a reluctance to perform concurrent surgery for fear that tumor cells may become established in the exposed areas of the resected prostate tissue. Previous studies have shown that concurrent surgery is safe in the presence of low-grade tumors without increasing the risk of prostate recurrence.

There is dogma about whether simultaneous TURBT and TURP may lead to increased recurrence rate in denuded areas of the resected prostate due to seeding and implantation of tumor cells, especially in the presence of high-grade UCB. Clinical data supporting this concern are lacking. Although not an uncommon situation in clinical practice [3], there is an ongoing debate about the oncologic safety of combining TURBT and TURP versus a staging procedure. However, TURP is unlikely to be considered in patients with extensive infiltrating bladder tumors, who may require or are likely to require cystectomy in the near future.

Recurrences are common in intermediate- and high-risk NMIBC. Several factors have been associated with an increased recurrence rate, including multifocality of the tumor, incomplete initial resection, continued exposure to urinary carcinogens in the presence of BOO, urothelial failure predisposing to de novo tumor formation, and seeding of tumor cells during transurethral resection has also been suggested as a possible mechanism [5,10,11]. Therefore, it may theoretically be beneficial to treat BOO in patients with UCB. Symptomatic BPH may play a role in the recurrence of UCB through several mechanisms. These include prolonged exposure to urinary carcinogens and retention, on the one hand, and bladder hyperplasia, hypoxia, and angiogenesis, which promote carcinogenesis on the other [10]. Therefore, facilitation of BOO could favor UCB recurrence by addressing and attenuating these carcinogenic mechanisms associated with BOO, which in most cases are secondary to BPH.

The implantation of urothelial cancer cells after endoscopic surgery has been demonstrated in several in vivo and in vitro studies, and in some clinical trials [4,5,12,13]. However, these data need to be interpreted carefully. Several of these studies involved open excisions, which have a high recurrences rate and therefore do not accurately reflect patients undergoing TURBT. Recent meta-analyzes showed no difference in tumor relapses between patients treated with simultaneous procedures and those who underwent TURBT alone [14]. It is unclear how many patients included in the studies had high-grade UCB. To our knowledge, this study is the first analysis to focus specifically on high-grade UCB. It shows no significant differences in prostatic urethra tumor recurrence between TURBT alone and simultaneous TURBT and TURP (7.3% in group 1 vs. 6.4% in group 2, p = 0.363).

Patients who underwent only TURBT were significantly more likely to have postoperative urinary retention due to BPH (8.8% vs. 3.6%, p = 0.044). Three of 13 patients in group 1 failed to void on multiple trials (<4 weeks) and underwent TURP at the time of the re-TURBT. Residual tumor found in the bladder at repeat resection tended to be lower in patients who underwent concurrent surgery than in the group that underwent TURBT only. However, they was not statistically significant (32.9% vs. 23.5%, p = 0.151). No tumor was found in the resected prostate tissue. In addition, it is important to consider that simultaneous TURBT and TURP reduce the need for additional hospitalization and the need for anesthesia compared to staged surgery, which may lead to more anesthetic complications in the elderly. Simultaneous procedures in geriatric patients have been shown to positively impact on patient’s quality of life [7]. These considerations must be weighed against the potential impossibility of perioperative chemotherapy with simultaneous TURBT and TURP.

Multivariable analysis of our data largely confirms existing findings. In particular, multifocality and tumor stage were associated with higher rates and shorter time to recurrence. In our analysis, a higher T stage was associated with a higher risk of prostate recurrence in both groups; this effect was more pronounced in the simultaneous surgery. TURBT alone, compared with TURBT and TURP, was not found to be an independent risk factor for prostate recurrence in the present study (odds ratio 0.86 [95% confidence interval]), confirming our previous findings [6].

Currently, it is traditionally considered that simultaneous TURBT and TURP in patients with low-grade UCB carry a low possibility for tumor implantation into the prostatic urethra [6]. Given anecdotal reports [12,13] that resection of a high-grade bladder tumors results in tumor seeding, many urologists avoid performing simultaneous TURBT and TURP. According to a recent systematic review and meta-analysis [15], it seems that simultaneous surgery could be considered in patients with high-grade tumors. The risk of bladder recurrence and prostatic urethra/fossa recurrences was 0.87 (95% CI 0.78–0.97) and 1.02 (95% CI 0.74–1.41), respectively. In our study, the overall bladder and prostatic urethra/fossa recurrence rate and time to recurrence and progression were similar in both groups.

Inherent limitations related to the retrospective nature of the analysis must be considered. In addition, the generalizability of the results may be limited by the restriction to a single institution and the small sample size. Another important consideration in interpreting these results is the lack of routine use of perioperative intravesical instillation in group 2. This limitation is mitigated by the fact that there are currently only data supporting perioperative chemotherapy for low-grade UCB, which were specifically excluded in the present analysis. Despite these limitations, this study fails to demonstrate a deleterious oncologic effect of the combination of TURP and TURBT for high-grade UCB in patients with symptomatic BPH.

## Conclusion

The present study suggests that in selected patients, simultaneous TURBT and TURP can be performed safely compared to TURBT alone, even when high-grade UCB is present.

## Abbreviations

Urothelial carcinoma of the bladder (UCB), carcinoma in situ (CIS), transurethral resection of bladder tumor (TURBT), non-muscle invasive bladder cancer (NMIBC), muscle-invasive bladder cancer (MIBC), International Prostate Symptom Score (IPSS)
